# NetGAM: Using generalized additive models to improve the predictive power of ecological network analyses constructed using time-series data

**DOI:** 10.1038/s43705-022-00106-7

**Published:** 2022-03-10

**Authors:** Samantha J. Gleich, Jacob A. Cram, J. L. Weissman, David A. Caron

**Affiliations:** 1 Department of Biological Sciences, University of Southern California, 3616 Trousdale Parkway, AHF, Los Angeles, CA 90089-0371 USA; 2 Horn Point Laboratory, University of Maryland Center for Environmental Science, 2020 Horns Point Road, Cambridge, MD 21613 USA

**Keywords:** Microbial biooceanography, Microbial ecology, Food webs

## Abstract

Ecological network analyses are used to identify potential biotic interactions between microorganisms from species abundance data. These analyses are often carried out using time-series data; however, time-series networks have unique statistical challenges. Time-dependent species abundance data can lead to species co-occurrence patterns that are not a result of direct, biotic associations and may therefore result in inaccurate network predictions. Here, we describe a generalize additive model (GAM)-based data transformation that removes time-series signals from species abundance data prior to running network analyses. Validation of the transformation was carried out by generating mock, time-series datasets, with an underlying covariance structure, running network analyses on these datasets with and without our GAM transformation, and comparing the network outputs to the known covariance structure of the simulated data. The results revealed that seasonal abundance patterns substantially decreased the accuracy of the inferred networks. In addition, the GAM transformation increased the predictive power (F1 score) of inferred ecological networks on average and improved the ability of network inference methods to capture important features of network structure. This study underscores the importance of considering temporal features when carrying out network analyses and describes a simple, effective tool that can be used to improve results.

## Introduction

Communities of microorganisms exist in virtually all natural environments on the planet and are shaped by complex interactions. Species-species interactions are diverse and may benefit both species involved (e.g., mutualism), only one (e.g., commensalism, parasitism, predation), or hurt both (e.g., competition) [1, 2]. Ecological network analyses are increasingly used by microbial ecologists to identify potential biotic interactions between organisms, and to form hypotheses regarding microbial community structure and function [1, 3, 4]. Commonly employed network-inference methods include pairwise correlation-based, regression-based, and probabilistic graphical methods [5, 6]. All of these methods leverage microbial abundance measurements to identify co-occurrence patterns between organisms [1, 7, 8]. Biotic interactions between organisms are then predicted based on the species’ co-occurrence patterns, resulting in a list of nodes (organisms) connected by edges (associations) [1, 7–9].

Abundance data that are used as input for ecological network analyses are often obtained through monthly time-series sampling efforts [10–15]. Time-series datasets are valuable because documenting changes in microbial community structure over long timeframes can provide information on the monthly, annual, or interannual variability in species abundances [16, 17]. For example, ~11 years of monthly sampling at the San Pedro Ocean Time-Series site revealed that 23% of bacterial operational taxonomic units demonstrated predictable, seasonal abundance patterns in surface waters [13]. Another time-series study that took place over ~6 years in the Western English Channel revealed that the month of year could explain over half of the variability in bacterial community composition [12]. Such studies highlight how long-term time-series datasets may be used to identify predictable changes in microbial community composition over time.

Time-series datasets can provide information on microbial community composition and structure, but ecological networks inferred from them should be built and interpreted with caution. There are statistical challenges associated with time-series network analyses because the samples are not independent over time [12, 18, 19]. This inherent time-dependence may be influenced in part by seasonally reoccurring patterns in the abiotic environment (e.g., seasonal mixing or upwelling events) or long-term changes in the environment over-time (e.g., rising seawater temperature) [17, 20, 21]. As a result of this time-dependence, species abundance patterns can lead to co-occurrence patterns that yield spurious network predictions [4, 22]. For instance, two species may both attain maximal abundances during spring nutrient upwelling events, even if no interactions occur between them. Their shared periodicity in this case may manifest itself as a false “association”. Time-dependence may also confound species co-occurrence patterns through the effects of Simpson’s paradox [23, 24]. If a mutualistic relationship exists between two species, one might expect a positive correlation between the abundances of the two species across samples. However, if the species respond differently to a third variable (e.g., month of year), then the positive association between the two species may be offset or reversed as a result of this time-dependence [24]. Such inaccurate associations indicate that caution should be exercised when carrying out network analyses on time-series datasets [7, 24].

Here, we propose and validate a generalized additive model (GAM)-based data transformation that corrects for potentially confounding time-series signals that are prevalent in microbial relative abundance data. The GAM transformation was conducted prior to carrying out ecological network analyses, in order to remove seasonal, long-term, and autocorrelative trends, thereby allowing researchers to focus on the residual statistical variability of the microbial abundance data. We contend that the residual variability is likely more indicative of true biotic associations than are untransformed data. We used GAMs in this data transformation method, as they are versatile, and commonly used to capture non-linear trends typical of time-series data [25]. Generalized additive models have been used to model both seasonal patterns and long-term trends in time-series data [25–27] and have also been used to capture autocorrelative signals [17, 28]. The GAM-based data transformation presented here has the potential to capture seasonal, long-term, and autocorrelative trends in time-series datasets, thus minimizing the influence that temporal signals have on inferred microbial co-occurrence patterns and increasing the F1 score of commonly employed networking methods.

## Materials and methods

Our general strategy was to compare the performance of four approaches for inferring microbial associations from abundance data with overlying time-series signals. The approaches were (1) pairwise spearman correlation analysis (SCC) [1, 29], (2) Graphical lasso analysis (Glasso) [30, 31], (3) pairwise SCC analysis with a pre-processing step where seasonal and long-term splines were fit to and subtracted from each variable using a GAM (GAM-SCC), and (4) Glasso with the same GAM subtraction approach (GAM-Glasso). Our validation strategy for the GAM transformation consisted of generating mock datasets with underlying associations, masking those associations by adding seasonal and long-term signals to the abundance data, and comparing the predicted associations obtained from each network inference method to the true species-species associations.

### Data simulation: generating mock abundance data with time-series properties

We generated mock abundance datasets that had a predetermined, underlying network structure and contained long-term and seasonal species abundance patterns. First, a covariance matrix was generated to describe the relationships between species in a mock dataset (Fig. S1, Panel 1). The covariance matrices were constructed with underlying network structures that followed either a scale-free Barabási-Albert model, a random Erdős-Rényi model, or a model of network topology based on a real microbial dataset (American Gut dataset; Fig. S1) [32, 33]. The Erdős-Rényi and Barabási-Albert model datasets were generated so that each dataset contained 400 species and 200 samples, and the American Gut datasets were created so that each dataset contained 127 species and 200 samples. A random Bernoulli distribution was used to simulate the covariance matrix for the Erdős-Rényi networks. We set the probability of interactions occurring between species in a given Erdős-Rényi network to 1%. The Barabási-Albert networks were generated using the “sample_pa” function in the igraph package [34]. The “graph2prec” function in the SpiecEasi package was used to predict the covariance matrix of the American Gut dataset [33]. The covariance between species in a dataset was considered “high” or “low” when the true associations in the covariance matrix were set to 100 or 10 respectively (Fig. S1, Panel 1). These covariance matrices describe the “real”, underlying species interactions in our mock datasets.

After generating a covariance matrix, the mean abundance for each species was generated from a normal distribution with a mean of 10 and a variance of 1. These mean abundance values and the covariance matrix were used to parameterize a multivariate normal distribution from which species abundance values for all 200 samples in a dataset were drawn (Fig. S1, Panel 2). The values generated from this multivariate normal distribution were the species abundance values without time-series features confounding the relationship between two associated species (Fig. S1, Panel 2).

“Gradual” or “abrupt” seasonal trends were added to 0%, 25%, 50% or 100% of the species in each mock dataset. The gradual seasonal trend increased over 5 months, peaked at a specific month, and decreased over 5 months. Conversely, the abrupt seasonal signal increased over 2 months, peaked at a specific month, and decreased over 2 months (Fig. S1, Panel 3). These seasonal signals were generated by plugging a vector of consecutive integers of length 200 (*N*_*t*_) into the gradual (Eq. (1)) or abrupt (Eq. (2)) seasonal equations (Fig. S1, Panel 3)…

1 $$Gradual:S_t = \left( {\frac{{\cos \left( {N_t \ast 2 \ast \frac{\pi }{{12}}} \right)}}{2}} \right) + 0.5$$

2 $$Abrupt:\,S_t = \left( {\left( {\frac{{\cos \left( {N_t \ast 2 \ast \frac{\pi }{{12}}} \right)}}{2}} \right) + 0.5} \right)^{10}$$

where *N* is the random vector of consecutive integers, *S* is the output seasonal vector, and *t* is the index of vectors *N* and *S*. The starting value of vector *N*_*t*_ was drawn at random for each species to allow the seasonal peaks to be centered at different months. Each element in the seasonal vector (*S*_*t*_) was then multiplied by the corresponding element in the abundance vector (*X*_*t*_) of a specific species to obtain mock species abundance values with a gradual or abrupt seasonal trend (Fig. S1, Panel 3).

A long-term time-series trend was added to the abundance values of 0% or 50% of the species in the mock datasets (Fig. S1, Panel 4). When a long-term signal was applied to 50% of the species in a dataset, half of the species were randomly selected to have this long-term trend. Then, a vector of linear values was generated following Eq. (3) such that…

3 $$Long - term\,trend:\,L_t = \pm m\left( {L_{t - 1}} \right) + 0.01$$

where *L*_*t*_ is the point in the line at the next time point and *m* is the slope of the line. The slope parameter (*m*) was generated from a random normal distribution with a mean of 0.01 and a variance of 0.01. The slope parameter (*m*) was also multiplied by −1 half of the time to ensure that half of the long-term trends increased over time and half decreased over time (Fig. S1, Panel 4). After generating the vector of linear values (*L*_*t*_), each element of this vector was added to each element of the abundance vector (*X*_*t*_) of a specific species to simulate long-term time-series trends (Fig. S1, Panel 4).

Time-series predictor columns were added to each dataset after applying monthly and long-term abundance trends to a portion of the species in the mock datasets. The predictors that were used in the downstream GAM-based data transformation were the month of the year (i.e., 1–12) and the day of the time-series (i.e., 1–200). In total, we generated 100 mock datasets for every combination of conditions (84 combinations total; Table S1), resulting in 8400 mock time-series datasets that were used in the downstream count data transformation, GAM subtraction, and network analysis procedures.

### Data simulation: Simulating count data from abundance values

The 8400 time-series datasets that were generated using the methods described above were transformed to make the abundance values resemble high-throughput sequencing data because microbial time-series sampling efforts are often processed using such molecular methods (e.g., tag-sequencing, meta-omics). Analysis of high-throughput sequencing data is complicated by the compositional (i.e., relative) nature of the data and by the high number of zeros that may be prevalent in a dataset (i.e., zero-inflation; see Supplementary Information) [35, 36]. Relative abundances of different species in natural communities are also highly skewed, so that relatively few species constitute most of the organisms in a sample although many rare species are also present [37, 38]. Therefore, species abundances were first exponentiated to increase the prevalence of abundant species and to decrease the prevalence of rare species (Fig. S1, Panel 5). The exponentiated species abundance values were then converted to relative abundance values by dividing each species count by the sum of all species counts in a sample (Fig. S1, Panel 6). The resulting relative abundance values and time-series predictor variables were used in data normalization and GAM-transformation steps prior to carrying out the network analyses.

### Network inference: Count data normalization and GAM transformation

**Fig. 1:**
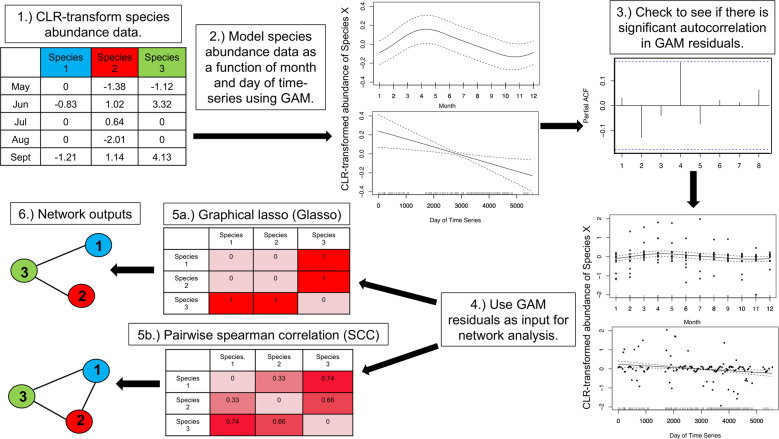
Steps used to carry out the GAM-based transformation of time-series species abundance data prior to carrying out pairwise spearman correlation (SCC) and graphical lasso (Glasso) ecological network analyses. The raw, species abundance data were first CLR-transformed (1). Generalized additive models (GAMs) were then fit to each species in the dataset (2) and the residuals of each GAM were checked for significant autocorrelation (3). The residuals of each GAM were extracted (4) and were used as input in the SCC and Glasso network analysis methods (5). Finally, the GAM-transformed network outputs were obtained (6; see text for additional details).

Several steps were taken to back out the species-species relationships in the mock datasets. We advocate these steps to infer network structure from a real time-series dataset. A centered log-ratio (CLR) transformation was first applied to the species relative abundance values to normalize the mock species abundance data across samples using the “clr” function in the compositions package in R (Fig. 1) [39]. This transformation step is important to avoid spurious inferences induced by the inherent compositionality of relative abundance data [31, 33, 36]. In addition to the CLR transformation used in our main network iterations, we carried out additional network iterations using the modified CLR [40], cumulative sum scaling [41], and total sum scaling [42] transformations (see Supplementary Information). In all cases, the normalized dataset was copied, with one copy subjected to a subsequent GAM transformation, and the other one not GAM-transformed.

**Fig. 2:**
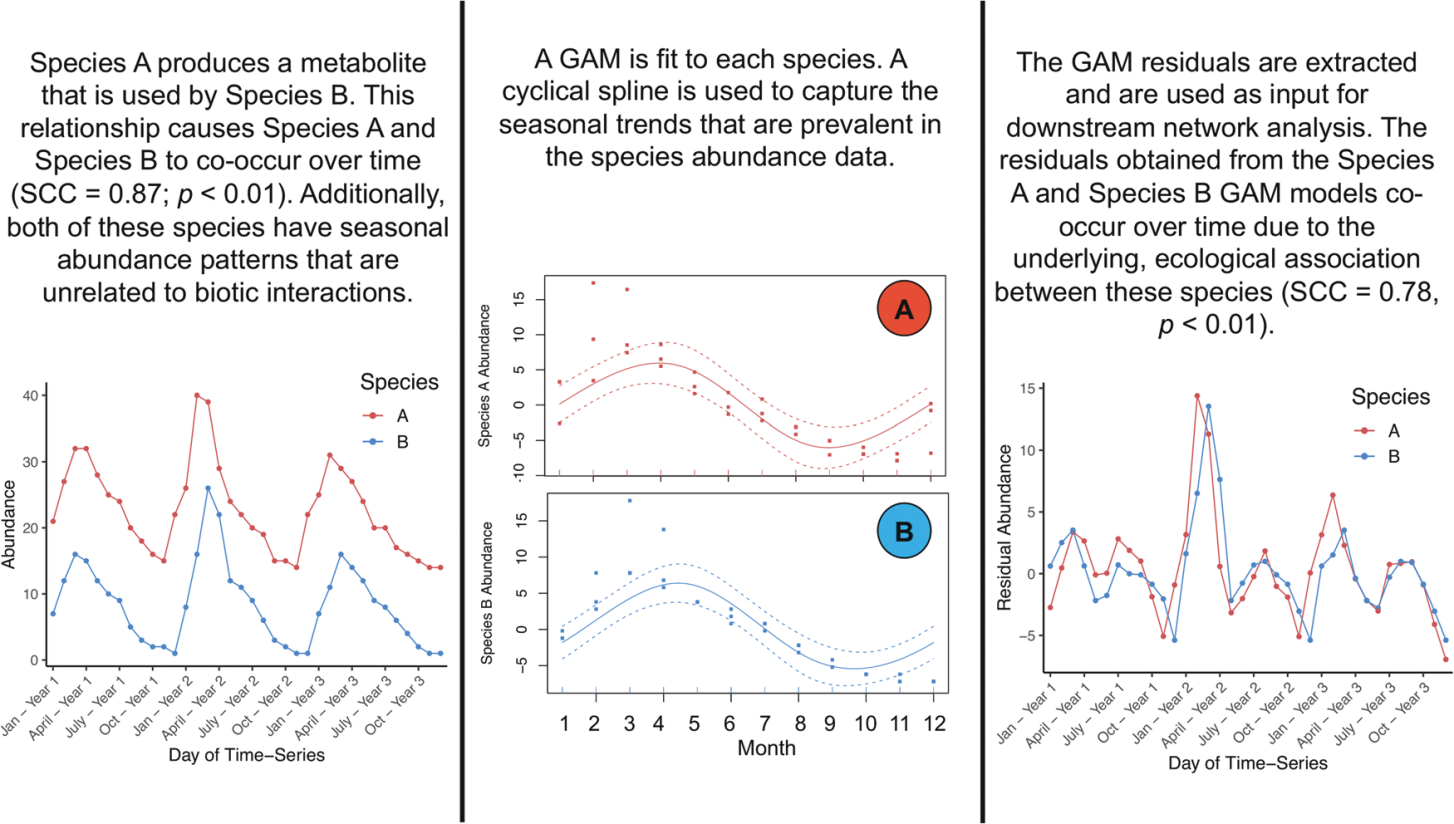
A conceptual figure that demonstrates how the GAM transformation can remove seasonal signals while preserving ecologically relevant species co-occurrence patterns. In this example, the co-occurrence pattern between Species A and Species B persists even after the seasonal signals are removed by the GAM transformation.

The GAM transformation was carried out by fitting GAMs to each individual species in the dataset to remove monthly signals, long-term trends, and autocorrelation from the species abundance data. These GAMs were fit using the “gamm” function in the mgcv package in R [43, 44]. The GAMs that were used included the “month of year” parameter as a cyclical spline predictor and the “day of time-series” parameter as a penalized thin-plate spline predictor (“ts” in the mgcv package; Fig. 1), which given our one-dimensional data is analogous to a natural cubic spline. In addition, the first GAM included a continuous AR1 (“corCAR1” in the mgcv package) correlation structure term in the model. This corCAR1 model was revised for specific species when the GAM could not be resolved or when significant autocorrelation was detected in the GAM residuals (Fig. 1). The GAM revision step fit 4 new GAMs with different correlation structure terms (i.e., “AR1”, “CompSymm”, “Exp”, and “Gaus”) to the species that could not be fit using the corCAR1 model or that contained significant autocorrelation in the corCAR1 GAM residuals. Then, the correlation structure term that addressed these issues for the largest number of individuals was used as the GAM model for this group of species. After fitting a GAM to all of the species in the input dataset, the residuals of each GAM were extracted and were used as the new, GAM-transformed abundance values (Fig. 1). These GAM residuals represent species abundance values with a reduced influence of time (Fig. 2) and were used as input in the downstream GAM-SCC and GAM-Glasso network analyses.

### Network inference: Network runs and statistical analyses

The pre-processed species abundance data with and without the GAM-removal of time-series signals were used in SCC and Glasso networks in order to compare the outputs of the SCC, GAM-SCC, Glasso, and GAM-Glasso network inference approaches (Fig. 1). Additional network iterations were also carried out using the CCLasso [45] and SPRING [40] network inference approaches (see Supplementary Information). For the SCC and Glasso network iterations, a nonparanormal transformation was applied to the species abundance datasets with and without the GAM transformation using the “huge.npn” function in the huge package in R [46]. Spearman correlation networks were then constructed by calculating the correlation between every pair of species in the mock abundance datasets. A Bonferroni-corrected *p* value of 0.01 was used as a cutoff to identify edges in these SCC networks. The Glasso networks were constructed by testing 30 regularization parameter values (i.e., lambdas) in each network using the “batch.pulsar” (criterion = “stars”; rep.num = 50) function in the pulsar package in R [47]. The lambda that resulted in the most stable network output was selected using the StARS method [48]. Finally, the graph that resulted from the StARS output was used to obtain a species adjacency matrix for the Glasso networks.

The species-species associations predicted by the SCC, GAM-SCC, Glasso, and GAM-Glasso networks were compared to the true species-species associations and the F1 scores of the network predictions were calculated. The F1 score is a measure of classification performance (presence or absence of an edge) that accounts for uneven classes, which is essential when dealing with sparse networks. The F1 scores of the GAM-transformed networks were compared to the networks that did not undergo GAM transformation using paired Wilcoxon tests with Bonferroni correction. An adjusted *p* value of 0.01 was used as a cutoff to identify under what circumstances the GAM significantly improved the F1 score of a Glasso or SCC network.

### Network inference: Comparison of predicted network structures

Additional networks were generated using the methods described above to compare the predicted network structures obtained from the GAM-Glasso, Glasso, GAM-SCC, and SCC approaches to the real network structures. These additional networks were constructed using smaller mock datasets to allow for better visualization of the network outputs and contained species with a gradual seasonal signal and high species-species covariance (see Supplementary Information). The average clustering coefficient and the degree distribution of these additional network outputs were calculated and used for the network structure comparisons. The average clustering coefficient of a network describes the likelihood that two species that are both associated with a third species are also associated with each other [49], and in a sense describes the “clumpiness” of a network. The network degree distributions describe the probability distribution of the number of interactions per node in a network [50].

## Results

### Seasonal abundance patterns decreased the performance of network inference methods

**Fig. 3:**
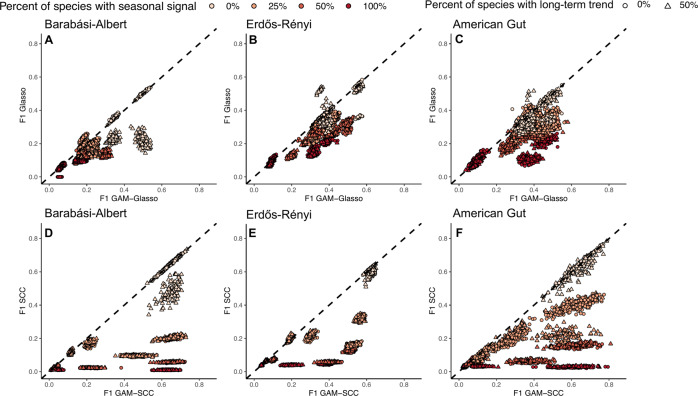
F1 scores of the networks constructed without GAM transformation (*y*-axis) plotted against the F1 scores of the GAM-transformed networks (*x*-axis) for all of the mock time-series datasets that were simulated. **A**–**C** Show the comparison between the Glasso and the GAM-Glasso networks, while (**D**–**F**) show the comparison between the SCC and the GAM-SCC networks. The dashed, black lines show the 1:1 relationship. Data points below the 1:1 line depict network outputs that had a higher F1 score after applying the GAM-based data transformation, while data points that fall above the 1:1 line depict network runs that had a higher F1 score without applying the GAM-based data transformation prior to network construction.

The F1 scores of the reconstructed network outputs generally decreased as the proportion of species in the mock dataset with a seasonal abundance pattern increased. The decreases in network F1 scores with increases in the percent of species with a seasonal abundance pattern were always prevalent in the Glasso and SCC networks when the GAM transformation was not applied and when some percentage of species (>0%) in the mock datasets had a seasonal abundance pattern (Fig. 3; Tables S2–S4). In general, the highest F1 scores were associated with networks that did not contain any species with an underlying seasonal signal (0%), while the lowest F1 scores were typically associated with networks in which all of the species had a seasonal abundance pattern (100%; Fig. 3; Tables S2–S4).

The general decline in network F1 score with a greater percentage of species exhibiting seasonality was often less pronounced when the mock datasets were GAM-transformed prior to carrying out the network analyses. For example, when species-species covariance was high, the GAM-SCC method tended to perform similarly regardless of whether 25%, 50%, or 100% of the species in a Barabási-Albert or American Gut dataset network had a gradual seasonal abundance pattern (Figs. S5 and S7, Panels B and F). The decline in network F1 score with increases in the percentage of species exhibiting seasonality was also less pronounced in GAM-Glasso networks when there was high covariance between species and when some of the species in the input dataset had a gradual, seasonal abundance pattern (Figs. S2–S4, Panels B and F).

### GAM transformation improved network inference on average

The GAM transformation increased the F1 score of the Glasso networks in 82.3% of all network runs (Fig. 3, Panels A–C; most points fall below the 1:1 line). Specifically, the GAM transformation significantly increased the mean F1 score of the Glasso networks when a gradual seasonal signal (Fig. S1, Panel 3) was applied to some fraction of the species in the input dataset (Figs. S2–S4, Panels B, D, F, and H; Tables S2–S4). The GAM-Glasso networks also had significantly higher F1 scores when 50% of species in the input dataset had an abrupt, seasonal abundance pattern (Figs. S2–S4, Panels A, C, E, and G; Tables S2–S4). For the Barabási-Albert models, the F1 scores of the GAM-Glasso networks were significantly greater than those of the Glasso networks when 50% of the species in the input dataset had a long-term trend with no (0%) seasonality (Fig. S2, Panels E–H; Table S2). Similar increases in F1 score were noted in the Erdős-Rényi and American Gut dataset, GAM-Glasso networks when the covariance between species was low and when 50% of the species in the dataset had long-term changes in abundance with no (0%) seasonality (Figs. S3–S4, Panels G and H; Tables S3–S4).

The GAM transformation also led to substantial increases in the F1 score of the SCC networks. Overall, the GAM transformation increased the F1 score of SCC networks in 78.2% of all network runs (Fig. 3, Panels D–F; most points fall below the 1:1 line). The average F1 scores of the networks were significantly greater when the data were GAM-transformed prior to carrying out a SCC network analysis when a gradual seasonal signal (Fig. S1, Panel 3) was applied to some fraction of the species in the input dataset (Figs. S5–S7, Panels B, D, F, and H; Tables S2–S4). The mean F1 scores of all GAM-SCC networks were also significantly greater than those of the SCC networks when there was high covariance between species and when an abrupt seasonal signal (Fig. S1, Panel 3) was applied to 25% or 50% of the species in the input dataset (Figs. S5–S7, Panels A and E; Tables S2–S4).

### GAM transformation improved the ability of Glasso and SCC networks to capture real network structure

**Fig. 4:**
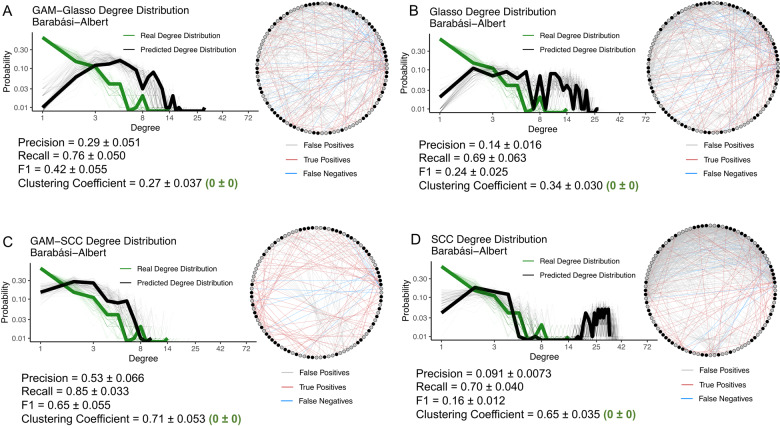
The GAM-SCC networks did the best job of capturing the real, Barabási-Albert network degree distribution. The degree distributions and network outputs of 100 GAM-Glasso (**A**), Glasso (**B**), GAM-SCC (**C**), and SCC (**D**) time-series networks that contained 100 species. The networks depicted were constructed with mock species abundance data that had an underlying Barabási-Albert network structure and that contained 50 species with a gradual, seasonal abundance pattern. The fine green lines on the log-log plots show the real degree distributions and the fine black lines show the network-predicted degree distributions. The bolded lines show the degree distributions of the representative networks that are depicted. On the representative network images, the red edges show those edges that are true positive associations, the blue edges show those edges that are false negative associations, and the gray edges show those edges that are false positive associations. The black nodes in the network images represent the species that have a seasonal abundance pattern, while the gray nodes represent those species that do not have a seasonal abundance pattern.

**Fig. 5:**
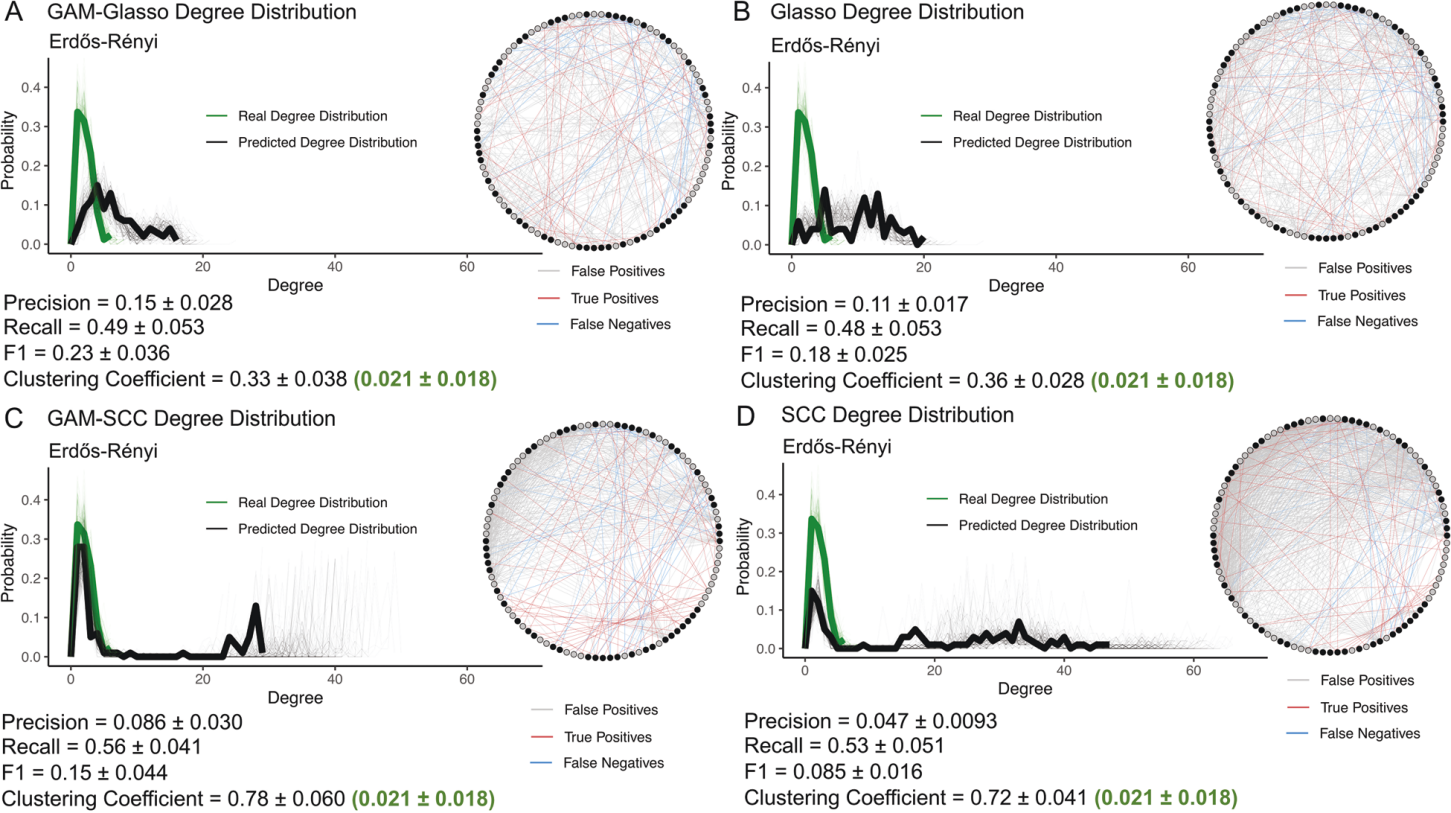
The GAM-Glasso networks did the best job of capturing the real, Erdős-Rényi network topology. The degree distributions and network outputs of 100 GAM-Glasso (**A**), Glasso (**B**), GAM-SCC (**C**), and SCC (**D**) time-series networks that contained 100 species. The networks depicted were constructed with mock species abundance data that had an underlying Erdős-Rényi network structure and that contained 50 species with a gradual, seasonal abundance pattern. Panels and color coding are the same as described for Fig. 4.

The GAM-transformation improved the ability of the Glasso and SCC methods to capture the underlying structure of the Barabási-Albert and Erdős-Rényi networks (Figs. 4–5). The real network degree distributions were more similar to the GAM-SCC and GAM-Glasso degree distributions than they were to the degree distributions of the SCC and Glasso networks without GAM transformation (Figs. 4–5). The GAM-SCC approach was the most successful in capturing the real, scale-free Barabási-Albert network degree distribution and had the highest average precision, recall, and F1 score of the four methods tested (Fig. 4; Panel C). Conversely, the GAM-Glasso approach did the best job of capturing the real, Erdős-Rényi network structure, as the SCC and GAM-SCC approaches predicted a number of high-degree nodes that were not present in the true network structures (Fig. 5; long right tail in Panels C and D). Some high-degree nodes were also predicted in the Glasso and GAM-Glasso, Erdős-Rényi networks (Fig. 5; long right tail in Panels A and B) but in general were less pronounced than those noted in the SCC and GAM-SCC degree distributions.

The average clustering coefficients of the GAM-Glasso networks were the most similar to the average clustering coefficients of the real networks, while the average clustering coefficients of the GAM-SCC networks were the least similar to those of the real networks (Figs. 4–5). The exaggerated average clustering coefficients of the SCC and GAM-SCC networks were to be expected, given the transitive nature of correlative relationships between variables. In general, the average clustering coefficient values that were obtained from the four network prediction approaches were substantially higher than the average clustering coefficient values of the real networks. These high, average clustering coefficient values implied that the outputs obtained from the network inference approaches resulted in networks that were “clumpier” than the real networks.

### Effectiveness of the GAM transformation decreased when seasonal abundance patterns were abrupt

The GAM transformation slightly decreased the predictive power of a small subset of the Glasso networks when abrupt seasonal abundance patterns (Fig. S1, Panel 3) were prevalent in the input dataset (Figs. S2–S4, Panels A, C, E, and G; Tables S2–S4). Specifically, when the covariance between species was high and all of the species (100%) in the input dataset had an abrupt seasonal abundance pattern, the F1 scores of the Glasso networks were significantly greater than those of the GAM-Glasso networks, though the magnitude of the differences in performance were minor (Figs. S2–S4, Panel A; Tables S2–S4). The GAM transformation also decreased or did not alter the F1 score for a small number of the SCC networks when abrupt seasonal abundance patterns were noted in the input dataset. There were some statistically significant, but very small, decreases in the GAM-SCC F1 scores relative to the SCC F1 scores when 50% of the species in the input dataset had long-term increases or decreases in abundance without having seasonal abundance patterns (0%; Figs. S5 and S7, Panels E–F).

In sum, there was never a substantial decrease, and there was often a substantial increase, in network F1 score when applying the GAM transformation to the data before network inference.

## Discussion

Ecological co-occurrence networks can be useful for capturing complex, biotic interactions when applied to high-throughput sequencing datasets of microbial communities. However, networks can yield inaccurate associations if time-series properties are prevalent in the dataset [4, 22]. The performance of the SCC and Glasso methods used in this study tended to decrease as the number of species with a seasonal abundance pattern in a dataset increased (Fig. 3; Tables S2–S4). This finding indicates that time-series networks can result in a higher number of false positive and false negative associations than networks that are constructed with datasets that lack time-series features. It is likely that the false positive associations that were detected in our time-series network outputs were indirect associations that resulted from the shared periodicity of two or more species over time. In addition, true species-species associations may have been missed by our network runs (false negatives) if seasonal and long-term signals overpowered the influence that other organisms had on species abundance patterns.

The GAM transformation carried out prior to network construction in this study improved network inference on average for all of the networking methods tested (Fig. 3; Fig. S10). The generally higher F1 scores that were noted following GAM transformation suggest that the GAM model was able to successfully capture and remove many of the seasonal and long-term signals that were prevalent in the mock communities. Previous efforts have been made to account for indirect or time-dependent associations in network analyses. For example, the EnDED program (environmentally driven edge detection) uses environmental variables (e.g., temperature, salinity, etc.) to predict and remove indirect, environmentally driven associations in an ecological network after network inference has already been performed, provided that environmental metadata are available [51]. Time-ordered networks have also been used by researchers to account for time when conducting network analyses [22, 52]. In time-ordered networks, a node is created for each species at each time point in a dataset, thus allowing those networks to capture associations between species at specific points in time [52]. However, this approach makes no correction for seasonal or long-term trends. The GAM-based data transformation proposed here provides an effective tool that can be used to account for multiple time-series features prior to carrying out ecological network analyses, can be tailored to a wide variety of time-series datasets, and can be used in conjunction with any downstream networking method.

The SCC and Glasso network comparisons carried out in this study demonstrated that both of these methods have unique strengths and weaknesses and that method selection may depend on the research question(s) being asked. With Barabási-Albert networks, the GAM transformation improved the F1 score and the degree distribution of the SCC networks more than those of the Glasso networks (Fig. 4). It is known that correlation-based networks tend to capture both direct and indirect associations in an input dataset [4, 9, 17], while Glasso networks can avoid capturing indirect associations [31]. The notably higher F1 scores and improvements in the degree distribution plots that were observed in the GAM-SCC networks relative to the SCC networks are presumably due to ability of the GAM to remove many of the indirect associations that would otherwise be detected in the SCC network analyses. While the GAM-SCC approach captured the real, Barabási-Albert degree distribution better than the GAM-Glasso approach, the GAM-Glasso approach was better able to capture the real, Erdős-Rényi degree distribution (Fig. 5). In addition, the average clustering coefficients of the GAM-Glasso networks were the most similar to the real, Barabási-Albert and Erdős-Rényi networks (Figs. 4–5), suggesting that the GAM-Glasso networks were more similar to the real networks than the SCC networks in terms of network “clumpiness”.

The GAM transformation did not substantively improve network inference under certain zero-inflation scenarios (Fig. S8) and when total sum scaling and cumulative sum scaling normalization methods were used as opposed to a CLR transformation (Fig. S9). The GAM transformation also led to decreases in F1 score at times when abrupt seasonal abundance patterns were prevalent (Figs. S2–-S7). It is possible that the smoothing functions used in the GAMs were unable to capture the some of the periodic spikes in species abundance that were noted in the abrupt, seasonal abundance patterns and therefore did not fully remove these abrupt signals. It is also possible that the GAM transformation inadvertently removed the influence that other species in the dataset had on the abundance pattern of a specific species and therefore decreased the number of true positive associations detected in the networks created using the GAM-transformed data. These scenarios highlight some potential issues that may arise if the GAM model used in the transformation overfits the data or does not accurately capture the seasonal and long-term signals that may be prevalent in the abundance data of a specific organism. If the GAM model overfits the data, then meaningful co-occurrence patterns between organisms may be lost in the GAM residuals. Conversely, if the GAM model fails to capture the time-series signals that may be influencing species abundance patterns, then the network predictions will still be influenced by temporal trends as opposed to true species-species associations. The observation that the GAM method did not always improve model performance when seasonal abundance patterns were abrupt suggests an opportunity for future improvement. Generalized additive models are good at fitting smooth trends in the data, but other methods might be better at removing abrupt seasonal signals. In any event, decreases in the F1 scores under these specific conditions (Figs. S2–S7) were marginal relative to the benefits obtained using the GAM transformation, suggesting that the GAM models used in our analyses were generally effective at removing time-series signals.

It may be beneficial to explore whether our GAM transformation improves the performance of other network analysis tools. Extended local Similarity Analysis (eLSA) which identifies time-lagged associations [53] and Liquid Analysis (LA) which explores interactions between trios of variables [54, 55] would likely be improved by removing seasonal signals. Future efforts may also be aimed at incorporating batch effects into our GAM framework to account for some of the additional non-biological factors that influence species abundance patterns over time. Generating a method that can identify sparse networks, time-lagged associations and three-way interactions, while removing seasonal signals and other sources of non-biological variability would be a clear future direction for a robust and flexible analysis of high-throughput data.

## Conclusion

The results of this study highlight the importance of considering temporal features when carrying out ecological network analyses with time-series data, given that time-dependent species abundance patterns may confound network predictions. The GAM-based data transformation presented here (NetGAM) provides a simple, yet effective tool that can be used to reduce the influence that time-series properties have on microbial abundance data prior to network construction. We published our method in a publicly available R package (https://github.com/sgleich/NetGAM) so that this data transformation can be used by other researchers in future time-series network analysis efforts. Accounting for seasonal abundance patterns, long-term trends, and autocorrelation in time-series datasets using our GAM method may substantially improve network inference. We recommend that future networking studies account for time-dependent species abundance patterns that may be prevalent in an input dataset in order to reduce the number of false positive and false negative associations that are detected through time-series network analyses.

## Supplementary information

Supplementary Information

Table S1

Table S2

Table S3

Table S4

Table S5

Table S6

Table S7
