# Supplementary material for: NetGAM: Using generalized additive models to improve the predictive power of ecological network analyses constructed using time-series data
Source: ISME Commun. 2022 Mar 10;2:23. doi: 10.1038/s43705-022-00106-7 (PMC9723797; doi:10.1038/s43705-022-00106-7)
Supplement: Supplementary file 1 — Supplementary Information [file 43705_2022_106_MOESM1_ESM.pdf]

Supplementary Information

Supplementary Materials and Methods

Network inference: Count data filtering

High-throughput sequencing data are at times filtered prior to carrying ecological network analyses due to the large size of these datasets and the high degree of zero-inflation that may be prevalent in these datasets [36]. In general, we recommend prefiltering a high-throughput sequencing dataset so that the species that are included in the GAM transformation and network analysis are present (i.e. non-zero) in a certain percentage of the samples. This type of prefiltering step will not only reduce the quantity of zero counts [36] but will also decrease the size of the dataset so that the transformation and network analysis procedures are less computationally intensive. There are also number of statistical methods that have been used to address zero-inflation specifically [35, 56-57], any of which could be used prior to carrying out the GAM transformation and network analyses proposed here.

Network inference: Comparison of predicted network structures (continued)

The network structure comparisons were carried out by generating 100 additional Barabási-Albert and Erdős-Rényi datasets with high species-species covariance. Each of the mock, time-series datasets that were used as input in these additional network runs contained 100 species and 200 samples. Additionally, 50% of the species in each of these datasets had a gradual, seasonal abundance pattern (Figure S1, Panel 3). The SCC, GAM-SCC, Glasso, and GAM-Glasso networking approaches were used to obtain network predictions for these extra network iterations and the average precision, recall, and F1 score obtained from each of the four approaches was calculated. The average clustering coefficients of the networks were also calculated and were compared to the average clustering coefficients of the real Barabási-Albert

and Erdős-Rényi networks. Finally, the degree distributions of the network runs were calculated and plotted alongside to the real network degree distributions.

Additional network iterations: Zero-inflated networks

We ran additional network iterations to determine whether the zero-inflation that may be prevalent in a real high-throughput sequencing dataset [35-36] would alter the effectiveness of the GAM transformation. To do this, we generated species abundance data in which 10%, 20%, 50% or 70% of the total counts in each dataset were set to zero. The zero-inflated datasets contained 400 species and 200 samples. Additionally, the covariance between species in the zero-inflated datasets was high (see Material and Methods) and the underlying covariance structure followed a Barabási-Albert model (Figure S1, Panel 1). Half of the species in each of the zero-inflated datasets had a gradual or abrupt seasonal signal (Figure S1, Panel 3) and half of the species in each of the zero-inflated datasets had a long-term increase or decrease in abundance (Figure S1, Panel 4). The methods used to generate these zero-inflated datasets follow the methods used to generate the other datasets in this study (see Materials and Methods); however, a zero-inflation step was added to the data simulation framework after exponentiating the species abundance data and before calculating the species relative abundances (i.e. Figure S1 between Panels 5 and 6).

The way in which we simulated our zero-inflated data may be representative of zero-inflation in a real high-throughput sequencing dataset if we assume that when an organism is less abundant in the environment, it is more likely show up as a zero count. Using this principle, we generated datasets in which we forced each species to have 20 zero counts (full dataset contains 10% zeros), 40 zero counts (full dataset contains 20% zeros), 100 zero counts (full dataset contains 50% zeros), and 140 zero counts (full dataset contains 70% zeros). In all cases, the

lowest abundance values for each species in each dataset were replaced with zeros. The zero-inflated simulations resulted in 100 mock datasets for every combination of conditions specified in Table S5. These zero-inflated datasets were CLR transformed and duplicated as described in the Materials and Methods. Then, one copy of each dataset was GAM-transformed and used to carry out GAM-Glasso and GAM-SCC network analyses. The second copy of each normalized dataset was not GAM-transformed and was used as input for the Glasso and SCC networking approaches. The F1 scores of the GAM-transformed, zero-inflated networks were compared to those zero-inflated networks that did not undergo GAM transformation in order to determine whether the GAM transformation improved network inference when species abundance data contained a high number of zeros.

Additional network iterations: Comparison of normalization methods

Additional network iterations were carried out to determine whether the method of count data normalization altered the performance of the GAM transformation. We tested 3 methods of normalization that are commonly used to analyze compositional data in addition to the CLR transformation from the compositions package in R [39] that was used in our main data transformation framework (see Materials and Methods). The additional data transformation methods that were used in our analyses were the modified CLR (MCLR) transformation from the SPRING package in R [40], the cumulative sum scaling (CSS) transformation from the metagenomeSeq package in R [41], and the total sum scaling (TSS) transformation from the NetCoMi package in R [42]. To determine whether the method of data normalization impacted the effectiveness of the GAM transformation, we generated additional mock species abundance datasets that contained 400 species and 200 samples. These datasets were parametrized to have high species-species covariance and the underlying network structure followed a Barabási-Albert

model (Figure S1, Panel 1; see Materials and Methods). Additionally, 50% of the species in each of these datasets had a gradual or abrupt seasonal abundance pattern (Figure S1, Panel 3) and 50% of the species in each of these datasets had a long-term trend in abundance over time (Figure S1, Panel 4). In total, 100 datasets containing species with a gradual seasonal signal and 100 datasets containing species with an abrupt seasonal signal were created and normalized (Figure 1, Panel 1) using the 4 normalization methods (CLR, MCLR, CSS, and TSS; Table S6). The SCC, Glasso, GAM-SCC, and GAM-Glasso network inferences were then carried out on each of these datasets (see Materials and Methods) and the network outputs were compared.

Additional network iterations: Comparison of network inference methods

There are a number of network inference methods that are commonly employed when carrying out ecological network analyses in addition to the Glasso and SCC methods used in our main network iterations. To test the effectiveness of the GAM transformation on some of these other network inference methods, we carried out additional network iterations using the SPRING [40] and CCLasso [45] programs. These methods were executed through the NetCoMi package in R [42]. The mock datasets that were used for these network runs contained 400 species and 200 samples. The datasets were generated so that there was high covariance between species (see Materials and Methods) and so that the networks had an underlying Barabási-Albert covariance structure (Figure S1, Panel 1). One hundred datasets were generated in which 50% of the species had a gradual seasonal signal (Figure S1, Panel 3) and 50% of the species had a long-term abundance trend (Figure S1, Panel 4). Additionally, 100 datasets were generated in which 50% of the species had an abrupt seasonal signal (Figure S1, Panel 3) and 50% of the species had a long-term abundance trend (Figure S1, Panel 4).

The 200 datasets that were generated were CLR-transformed and duplicated. Then, one copy of each dataset was GAM transformed (see Materials and Methods). Both the CLR transformation and the GAM transformation used in our network iterations introduce negative values into a species abundance dataset. These negative values are incompatible with the CCLasso and SPRING network inference methods, as these methods take count data as input and therefore require that an input dataset contain only values greater than or equal to 0. To address this incompatibility issue, we calculated the minimum value in each of our transformed mock datasets and added the absolute value of that minimum to every count in the dataset. Adding these pseudo-counts produced datasets with and without the GAM transformation that contained values greater than or equal to 0. These pseudo-count datasets were used as input for the 4 network inference methods (Glasso, SCC, SPRING, and CCLasso), resulting in 8 network outputs (Glasso, GAM-Glasso, SCC, GAM-SCC, SPRING, GAM-SPRING, CCLasso, GAM-CCLasso; Table S7). The network outputs with GAM transformation were compared to those without GAM transformation to determine the effectiveness of the GAM when paired with each network inference method.

Supplementary Results

Zero-inflation impacted the performance of the GAM transformation

The way in which zero-inflation impacted the GAM transformation depended on the number of zeros in the input dataset, the type of seasonal signal prevalent in the input dataset, and the network inference method used. When half of the species in an input dataset had a gradual seasonal signal and 10% or 20% of the input dataset was set to zero, the F1 scores of the GAM-Glasso networks were significantly higher than those of the Glasso networks (Figure S8,

Panel A; Table S5). Conversely, the F1 scores of the GAM-Glasso networks were significantly lower than those of the Glasso networks when gradual seasonal abundance patterns were prevalent and when 50% or 70% of the input dataset was set to zero (Figure S8, Panel A; Table S5). The network iterations with abrupt seasonal abundance patterns revealed that the GAM-Glasso network F1 scores were significantly higher than those of the Glasso networks when 20% of the input dataset was forced to zero (Figure S8, Panel B; Table S5). The Glasso networks constructed with datasets containing abrupt seasonal abundance patterns also revealed that the GAM-transformed network F1 scores were significantly lower than the F1 scores of the Glasso networks without GAM transformation when 50% or 70% of the dataset was forced to zero (Figure S8, Panel B; Table S5).

The F1 scores of the GAM-SCC zero-inflated networks were always significantly greater than the F1 scores of the SCC zero-inflated networks when a gradual seasonal abundance pattern was prevalent in the input dataset (Figure S8, Panel C; Table S5). However, when the input dataset contained species with an abrupt seasonal abundance pattern and when 10% or 20% of the input dataset was forced to zero, the F1 scores of the GAM-SCC networks were significantly lower than those of the SCC networks (Figure S8, Panel D; Table S5). The effectiveness of the GAM was apparent when abrupt seasonal abundance patterns were prevalent and when 50% or 70% of the dataset was forced to zero, as the GAM-SCC network F1 scores were significantly greater than those of the SCC networks under these circumstances (Figure S8, Panel D; Table S5).

CLR and MCLR normalization methods were the most effective with the GAM-transformation

The 4 normalization methods (CLR, MCLR, CSS, and TSS) resulted in very different Glasso, GAM-Glasso, SCC, and GAM-SCC network F1 scores. The GAM-SCC and the GAM-

Glasso networks that were constructed with the CLR or MCLR-normalized datasets had significantly higher F1 scores than the SCC and Glasso networks that did no undergo GAM transformation (Figure S9; Table S6). Conversely, the CSS and TSS normalization methods were typically not effective in combination with the GAM transformation. The F1 scores of the GAM-Glasso networks that were constructed with the CSS and TSS-normalized datasets were significantly lower than the F1 scores of the CSS and TSS-normalized Glasso networks (Figure S9, Panels A and B; Table S6). The GAM-SCC networks constructed with the CSS-normalized datasets also had significantly lower F1 scores than the corresponding CSS-normalized SCC networks (Figure S9, Panels C and D; Table S6). Importantly, the mean F1 scores of the CLR-normalized GAM-Glasso and GAM-SCC networks were always greater than those of the CSS and TSS-normalized networks that did not undergo GAM transformation, demonstrating that the CLR normalization method in combination with the GAM transformation yielded the most accurate network predictions under all of the tested circumstances (Table S6). Given these observations, we recommend applying a CLR transformation to species relative abundance data prior to carrying our GAM-based data transformation.

GAM transformation improved network inference regardless of what networking method was used

The F1 scores of the network analyses carried out with GAM-transformed data were significantly greater than those that were not GAM-transformed, regardless of what network inference method was used (Figure S10; Table S7). When a gradual seasonal signal was applied to 50% of the species in an input dataset, the GAM transformation led to substantial increases in the F1 scores obtained from all of the network inference methods that were used in our analyses (Figure S10, Panel A). The higher F1 scores that were observed in the GAM-transformed

networks were less pronounced when 50% of the species in the input dataset had an abrupt seasonal abundance pattern; however, the F1 scores of the GAM-transformed networks with an abrupt seasonal signal were still significantly greater than those networks that were constructed without the GAM transformation (Figure S10, Panel B).

References

56. Martín-Fernández J-A, Hron K, Templ M, Filzmoser P, Palarea-Albaladejo J. Bayesian-multiplicative treatment of count zeros in compositional data sets. *Stat. Modelling*. 2015;15(2):134-58.

57. Palarea-Albaladejo J, Martín-Fernández J-A. A modified EM alr-algorithm for replacing rounded zeros in compositional data sets. *Comput. Geosci*. 2008;34(8):902-17.

Supplementary Figures

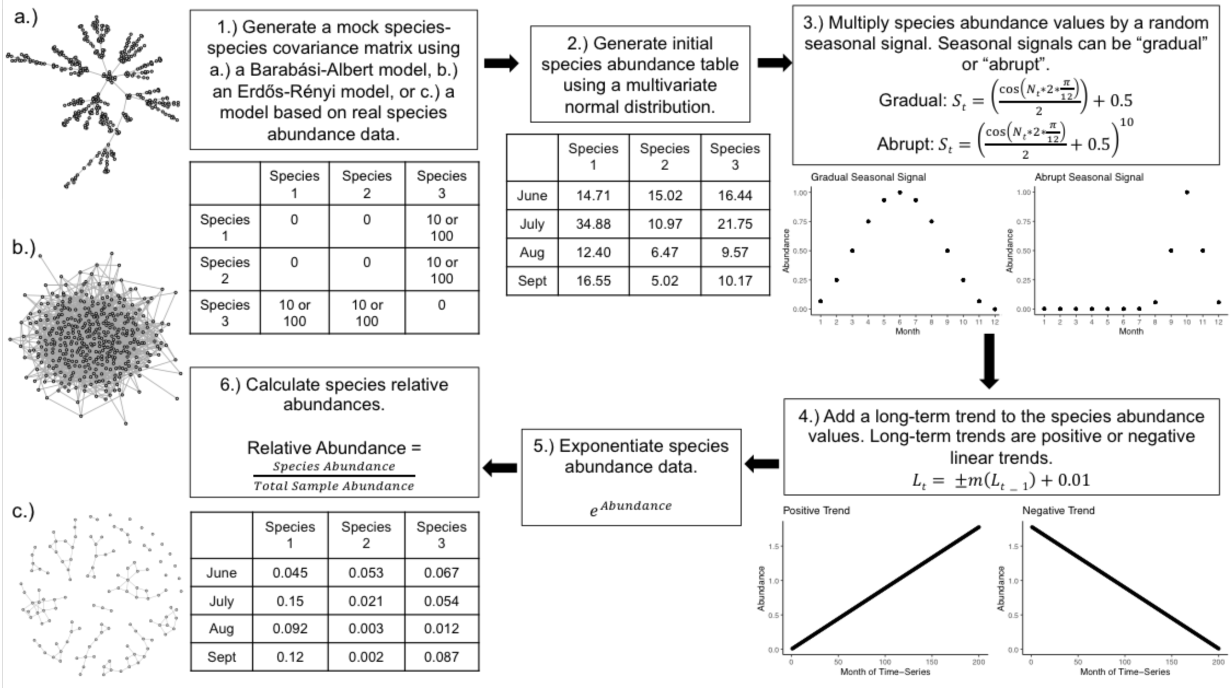

Figure S1: Steps used to generate mock species abundance data with seasonal signals, long-term trends, and an underlying covariance structure. The networks labeled a-c show the 3 network structures that were tested in the GAM transformation method validation—the Barabási-Albert network (a), the Erdős-Rényi network (b), and the network predicted from the American Gut dataset (c).

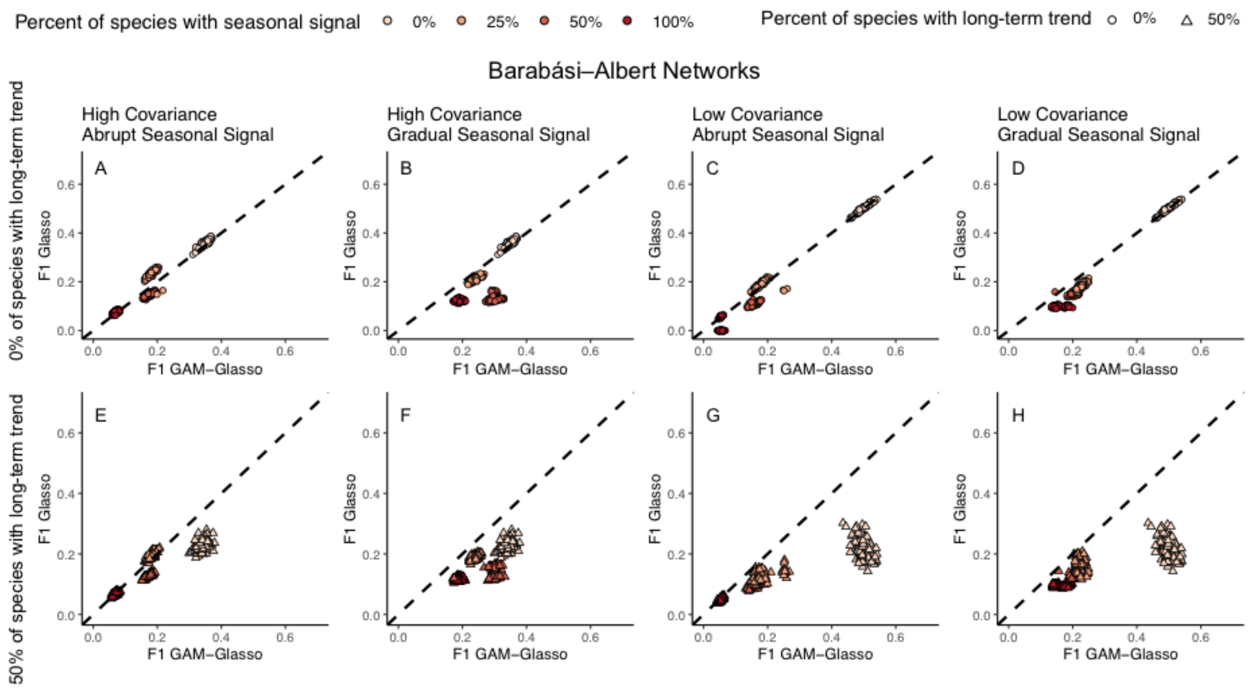

Figure S2: F1 score of the Glasso networking method without the GAM data transformation (F1 Glasso) as a function of the F1 score of the Glasso networking method with the GAM data transformation (F1 GAM-Glasso) for datasets in which the real network structure was that of a Barabási–Albert model. The results of 400 networks are shown in each panel. The species abundance data used in panels A-D did not have any long-term trends, while the species abundance data used in panels E-H had long-term trends added to 50% of the network species. The dashed, black lines show the 1:1 relationship. Data points below the 1:1 line depict network runs that had a higher GAM-Glasso F1 score, while data points that fall above the 1:1 line depict network runs that had a higher Glasso F1 score.

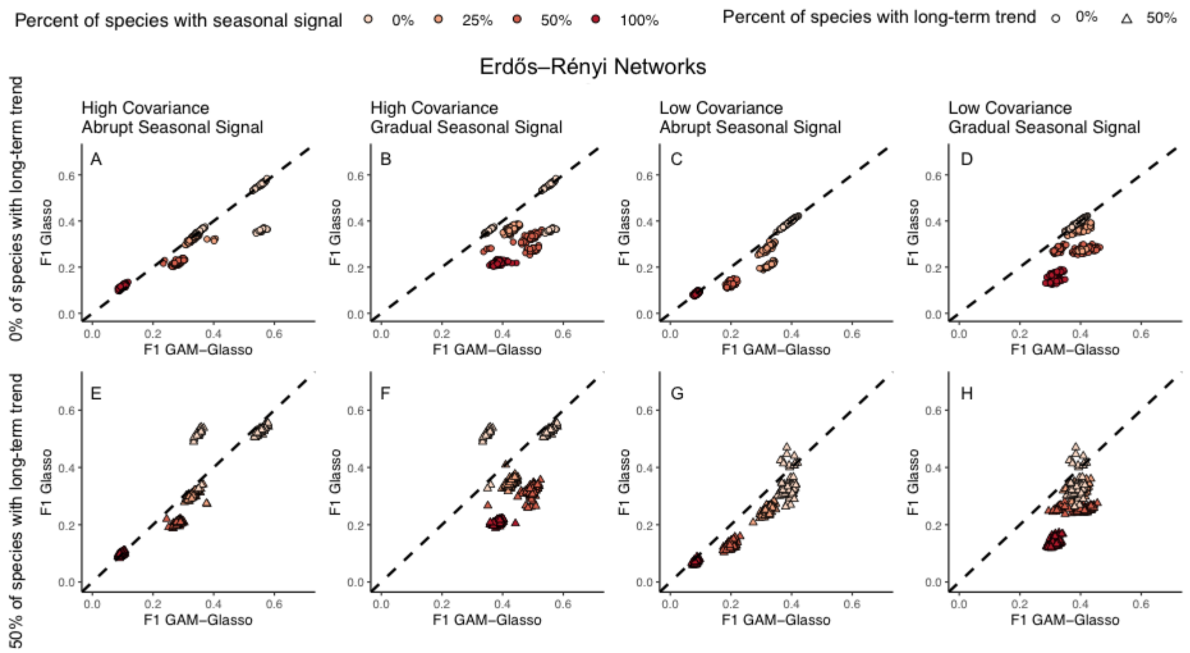

Figure S3: F1 score of the Glasso networking method without the GAM data transformation (F1 Glasso) as a function of the F1 score of the Glasso networking method with the GAM data transformation (F1 GAM-Glasso) for datasets in which the real network structure was that of an Erdős-Rényi model. Panels and details are the same as in Figure S2.

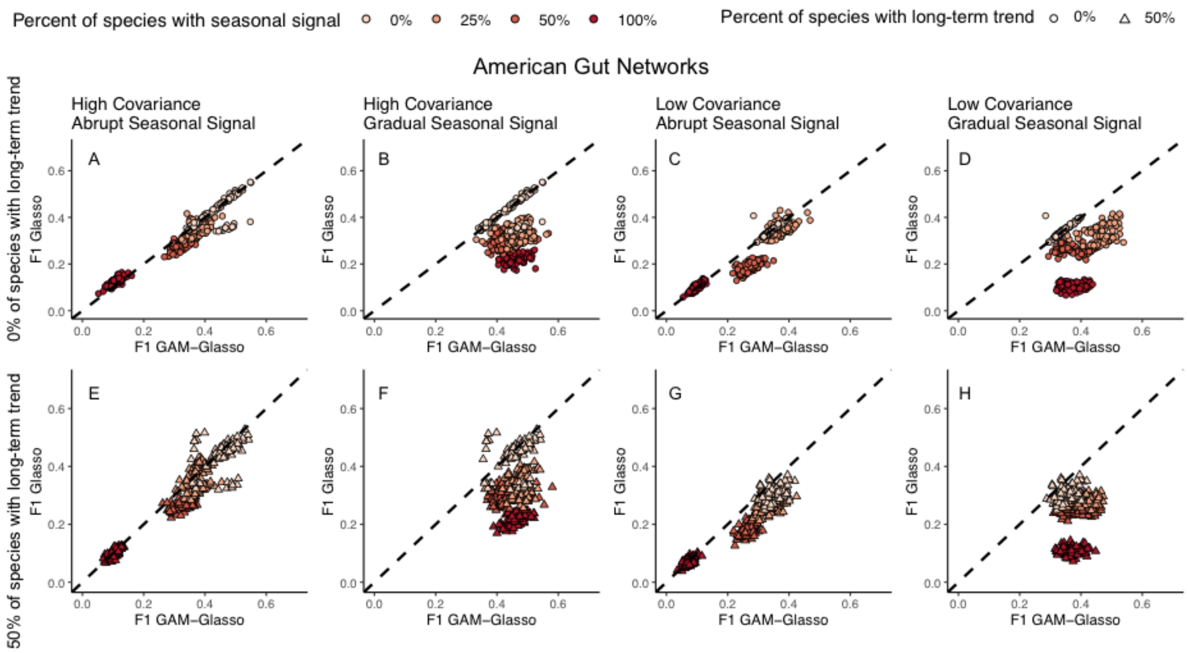

Figure S4: F1 score of the Glasso networking method without the GAM data transformation (F1 Glasso) as a function of the F1 score of the Glasso networking method with the GAM data transformation (F1 GAM-Glasso) for datasets in which the real network structure was estimated from the American Gut dataset. Panels and details are the same as in Figure S2.

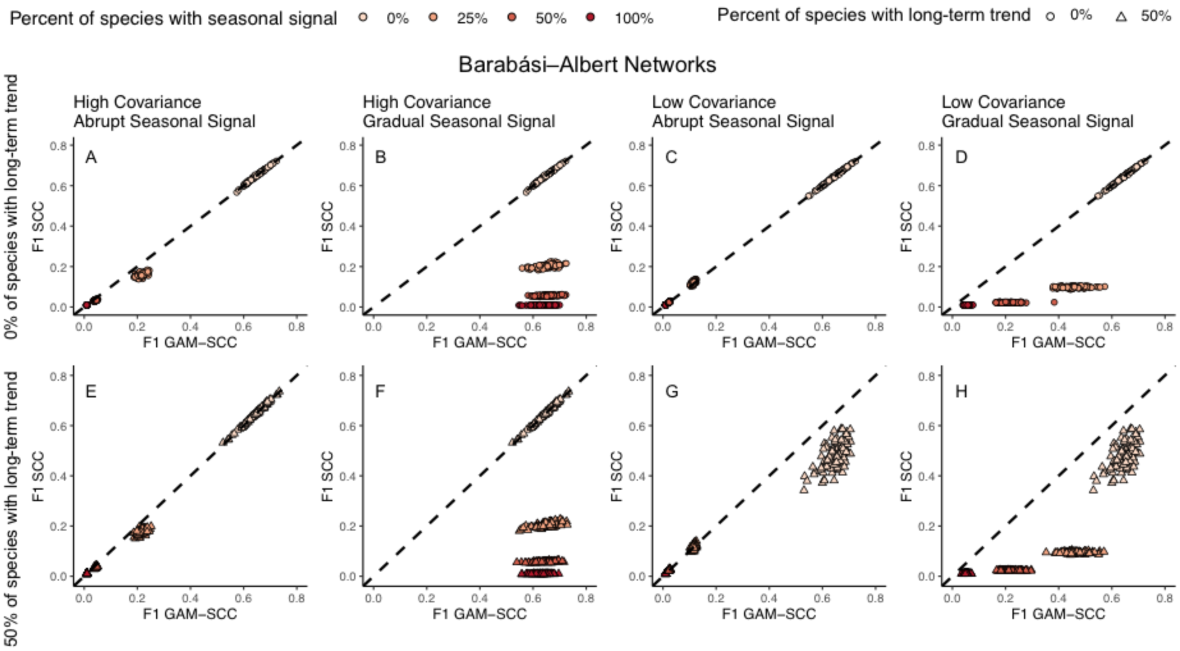

Figure S5: F1 score of the SCC networking method without the GAM data transformation (F1 SCC) as a function of the F1 score of the SCC networking method with the GAM data transformation (F1 GAM-SCC) for datasets in which the real network structure was that of a Barabási–Albert model. Panels and details are the same as in Figure S2.

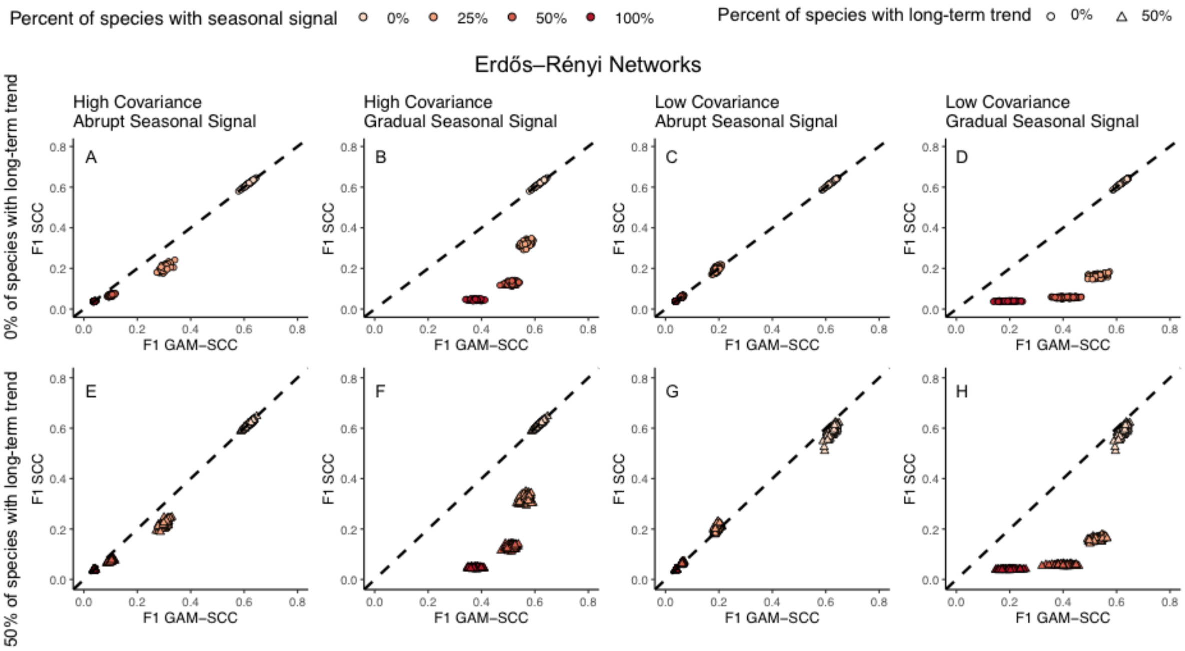

Figure S6: F1 score of the SCC networking method without the GAM data transformation (F1 SCC) as a function of the F1 score of the SCC networking method with the GAM data transformation (F1 GAM-SCC) for datasets in which the real network structure was that of an Erdős-Rényi model. Panels and details are the same as in Figure S2.

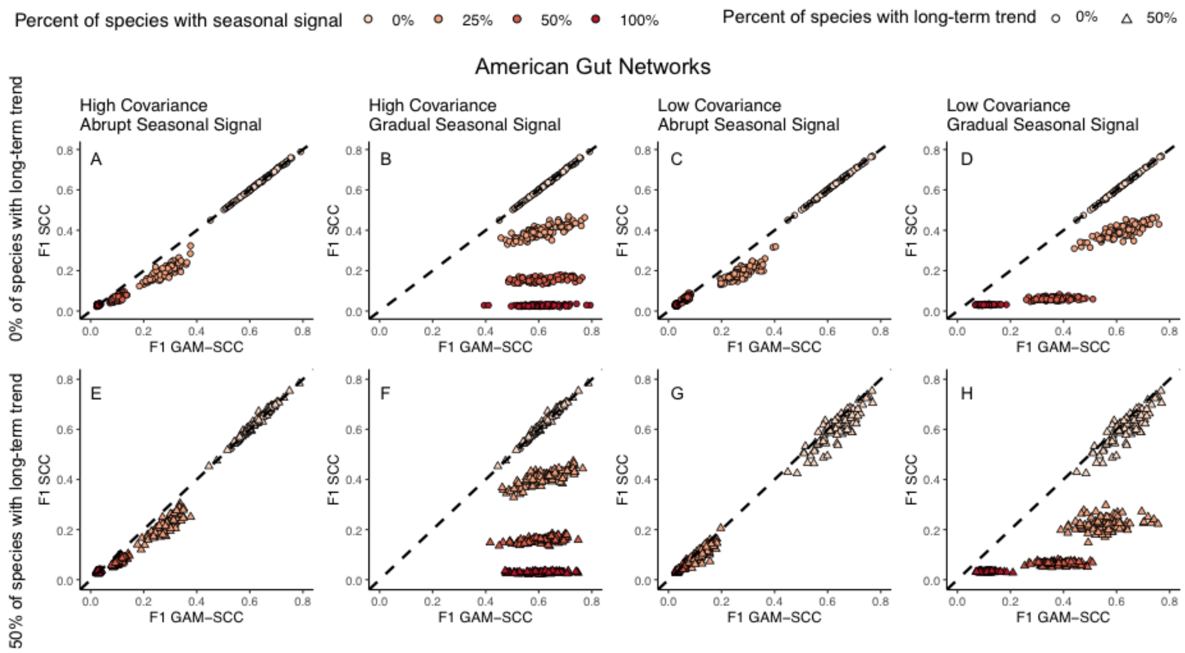

Figure S7: F1 score of the SCC networking method without the GAM data transformation (F1 SCC) as a function of the F1 score of the SCC networking method with the GAM data transformation (F1 GAM-SCC) for datasets in which the real network structure was estimated based on the American Gut dataset. Panels and details are the same as in Figure S2.

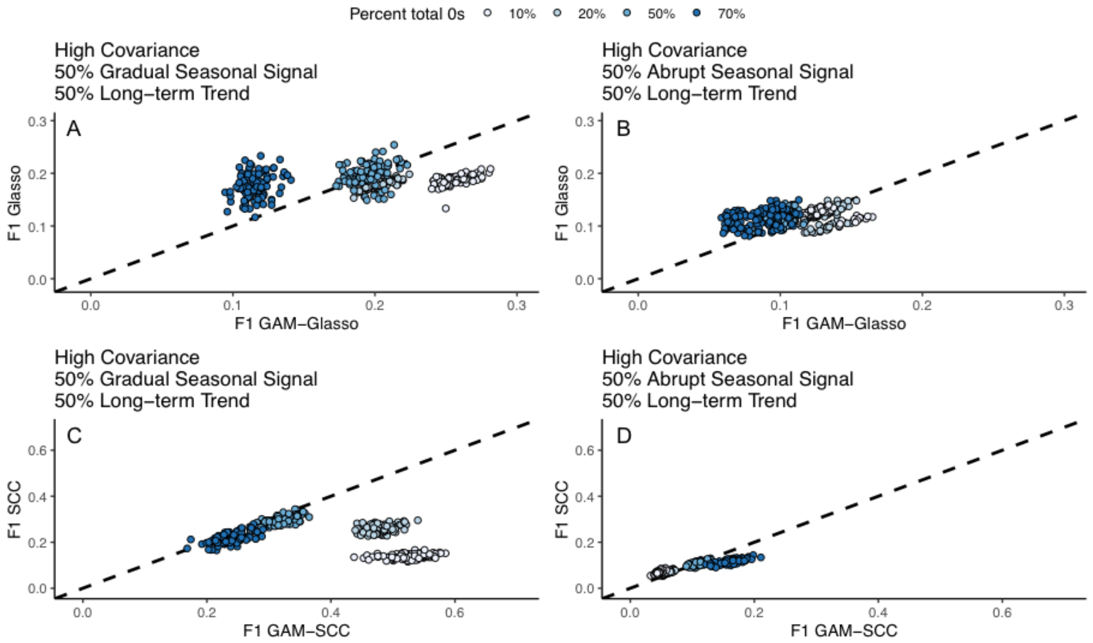

Figure S8: F1 score of the Glasso (Panels A and B) and SCC (Panels C and D) networking methods without the GAM data transformation (F1 Glasso and F1 SCC) as a function of the F1 score of these networking methods with the GAM data transformation (F1 GAM-Glasso and F1 GAM-SCC) for zero-inflated datasets in which a certain percentage of the datapoints for each species were set to zero. The datasets used in these network iterations had an underlying Barabási–Albert network structure and contained species with either a gradual seasonal signal and a long-term trend (Panels A and C) or an abrupt seasonal signal and a long-term trend (Panels B and D). The dashed, black lines show the 1:1 relationship. Data points below the 1:1 line depict network runs that had a higher GAM-transformed F1 score, while data points that fall above the 1:1 line depict network runs that had a higher F1 score without the GAM transformation.

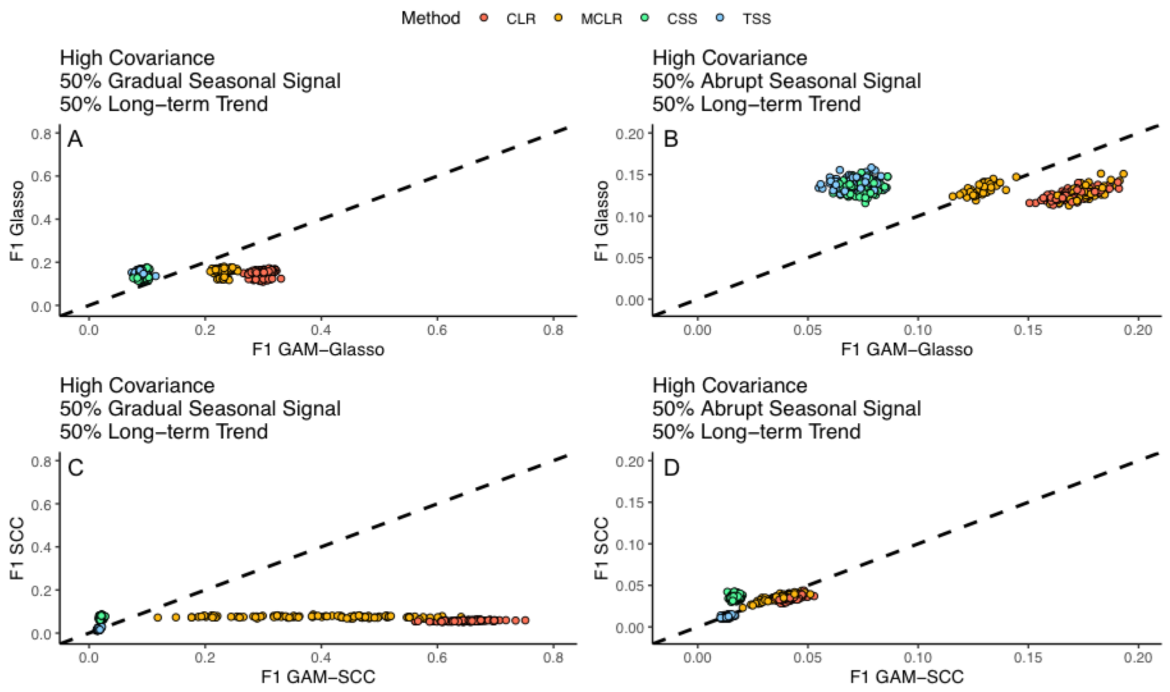

Figure S9: F1 score of the Glasso (Panels A and B) and SCC (Panels C and D) networking methods without the GAM data transformation (F1 Glasso and F1 SCC) as a function of the F1 score of these networking methods with the GAM data transformation (F1 GAM-Glasso and F1 GAM-SCC) for datasets that were normalized using 4 different normalization techniques. The datasets used in these network iterations had an underlying Barabási–Albert network structure and contained species with either a gradual seasonal signal and a long-term trend (Panels A and C) or an abrupt seasonal signal and a long-term trend (Panels B and D). The dashed, black lines show the 1:1 relationship. Data points below the 1:1 line depict network runs that had a higher GAM-transformed F1 score, while data points that fall above the 1:1 line depict network runs that had a higher F1 score without the GAM transformation.

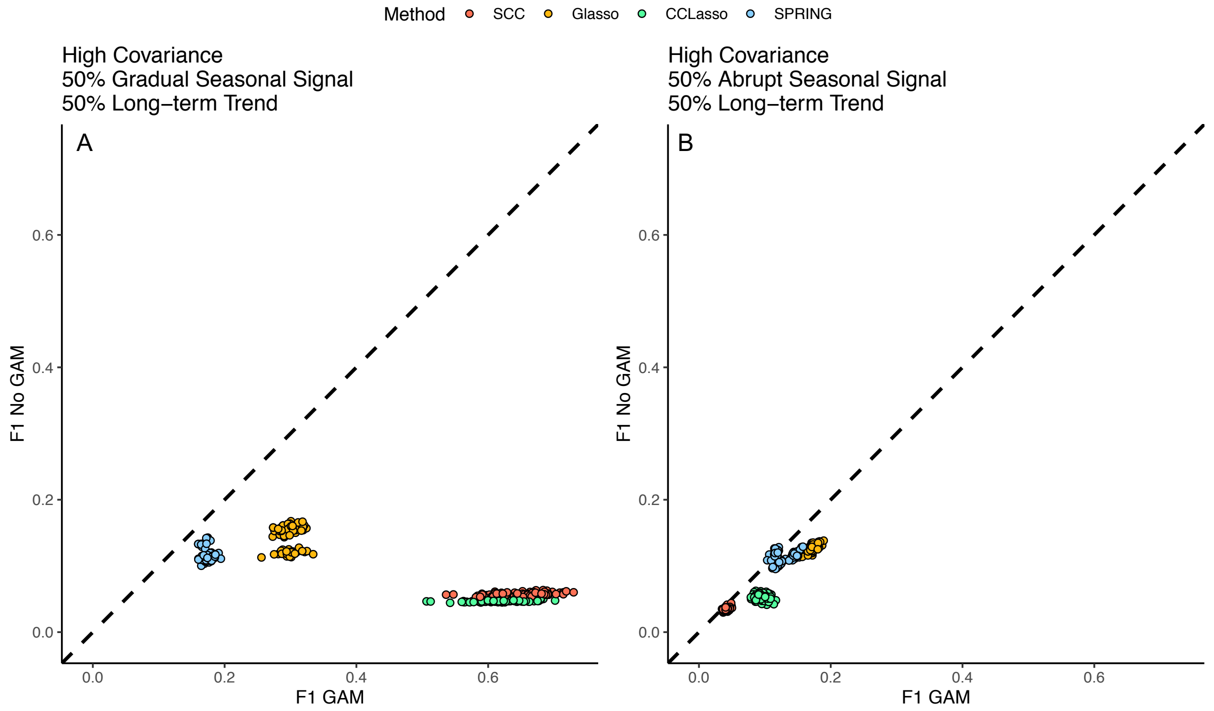

Figure S10: F1 score of networks obtained from 4 networking methods without the GAM data transformation as a function of the F1 score of these networking methods with the GAM data transformation. The datasets used in these network iterations had an underlying Barabási–Albert network structure and contained species with either a gradual seasonal signal and a long-term trend (Panel A) or an abrupt seasonal signal and a long-term trend (Panel B). The dashed, black lines show the 1:1 relationship. Data points below the 1:1 line depict network runs that had a higher GAM-transformed F1 score, while data points that fall above the 1:1 line depict network runs that had a higher F1 score without the GAM transformation.

Supplementary Table Legends

Table S1: Conditions used to generate the mock, time-series datasets that were used in this study. For each condition (row), 100 mock datasets were created. Then, the 4 (Glasso, GAM-Glasso, SCC, and GAM-SCC) network analysis approaches were used to infer species-species associations from each mock dataset. In total, 8 400 mock datasets were created, and 33 600 networks were generated (8 400 * 4).

Table S2: Summary statistics for the Glasso, GAM-Glasso, SCC, and GAM-SCC networks that were generated from mock datasets with a Barabási-Albert network structure.

Table S3: Summary statistics for the Glasso, GAM-Glasso, SCC, and GAM-SCC networks that were generated from mock datasets with an Erdős-Rényi network structure.

Table S4: Summary statistics for the Glasso, GAM-Glasso, SCC, and GAM-SCC networks that were generated from mock datasets with a network structure that was estimated from the American Gut dataset.

Table S5: Summary statistics for the Glasso, GAM-Glasso, SCC, and GAM-SCC networks that were constructed using mock datasets that contained varying degrees of zero-inflation (10%-70%).

Table S6: Summary statistics for the Glasso, GAM-Glasso, SCC, and GAM-SCC networks that were constructed after normalizing mock datasets using 4 different methods (CLR, MCLR, CSS, and TSS).

Table S7: Summary statistics for Glasso, GAM-Glasso, SCC, GAM-SCC, CCLasso, GAM-CCLasso, and SPRING, GAM-SPRING additional network runs.
